# Myocardial blood flow in patients with sepsis

**DOI:** 10.14814/phy2.70605

**Published:** 2025-10-06

**Authors:** Peter Andreas Christiansen, Farnoosh Nedaei, Casper Sejersen, Hannah G. Caldwell, Lasse Gliemann, Anne Sofie Andreasen, Christian Søborg, Henrik Segelcke Thomsen, Jakob M. Møller, Per Lav Madsen

**Affiliations:** ^1^ The August Krogh Section for Human Physiology, Department of Nutrition, Exercise and Sports University of Copenhagen Copenhagen Denmark; ^2^ Department of Cardiology Herlev‐Gentofte Hospital, University of Copenhagen Copenhagen Denmark; ^3^ Department of Intensive Care Herlev‐Gentofte Hospital Herlev Denmark; ^4^ Department of Infectious Diseases Herlev‐Gentofte Hospital Herlev Denmark; ^5^ Department of Radiology Herlev‐Gentofte Hospital Herlev Denmark

**Keywords:** magnetic resonance imaging, myocardial blood flow, organ blood flow, sepsis, septic shock

## Abstract

The high mortality of systemic infection results from its associated cardiovascular depression. Cardiac depression typically normalizes if the patient recovers from the infection, but recent studies suggest that these patients have an increased long‐term risk of developing cardiovascular disease after the septic episode. These findings have reignited interest in sepsis‐associated cardiac function and myocardial blood flow, which remain poorly understood in humans. We studied cardiac function and myocardial microvascular perfusion using gadolinium‐contrast magnetic resonance imaging in a cohort of patients during the initial recovery period of sepsis (*n* = 16) or septic shock (*n* = 5) and after full recovery 1–2 months later (*n* = 13). In addition, hepatic, splenic, and renal cortical perfusion were also assessed. With infection, cardiac output (+27%), the rate‐pressure‐product (+21%), and the left ventricle (LV) peak‐ejection (+36%) and peak‐filling (+35%) rates increased compared to full recovery (all *p* < 0.05). Onset of LV myocardial perfusion and the time to peak LV myocardial perfusion of gadolinium‐contrast occurred earlier during initial than after recovery, with a numerically higher LV myocardium wash‐in rate (23 ± 22 vs. 14 ± 14 s^−1^; *p* = 0.07). LV myocardial fibrosis was not seen in any patients. Renal cortical, splenic, and hepatic perfusion parameters including onset, time‐to‐peak, and wash‐in rates of gadolinium contrast were comparable between initial and full recovery, except for a lower hepatic wash‐in rate during initial recovery (13 ± 10 vs. 22 ± 16 s^−1^; *p* = 0.03). Our study supports that myocardial microvascular dysfunction is unlikely to contribute to cardiovascular disease after severe infection. Conversely, hepatic hypoperfusion during sepsis may explain the commonly observed hepatic dysfunction in sepsis.

## 1 INTRODUCTION

The high mortality of severe systemic infection is largely attributed to its cardiac and circulatory impact (Angus & van der Poll, 2013; Court et al., 2002; Kakihana et al., 2016; Vallabhajosyula et al., 2021). Sepsis and septic shock are associated with myocardial depression, as patients often present with a low left ventricle ejection fraction (LVEF) in the acute phase, that however usually normalizes within days if only the infection is successfully treated (Bouhemad et al., 2008; Court et al., 2002). Recent studies, however, suggest that sepsis‐induced cardiac dysfunction and ventricular remodeling may persist (Greer, 2015; Lv & Wang, 2016), and epidemiological studies further associate a septic episode with a sustained higher risk of cardiovascular disease, including heart failure, years after the initial episode (Grebenchikov & Kuzovlev, 2021; Kosyakovsky et al., 2021; Lin et al., 2022). These recent findings have reignited an interest in the mechanistic links from sepsis to cardiovascular disease, which remain poorly characterized in humans.

Although the impact of acute sepsis on myocardial blood flow, which is critical for the later development of heart failure, is not well understood in humans, certain mechanisms are well‐established. On the one hand, the decrease in cardiac contractile reserves during sepsis and septic shock is not simply a consequence of significant global myocardial ischemia (Adams et al., 1990; Merx & Weber, 2007). Instead, myocardial depression is related to circulating myocardial depressant factors for which potential candidates among others include cytokines, prostanoids, and nitric oxide with influence also from endothelial activation and induction of the coagulation cascade (Merx & Weber, 2007). On the other hand, microvasular dysfunction is still suggested to be a motor of the cardiovascular compromise (Antonucci et al., 2014; Merx & Weber, 2007). While human studies on microcirculatory changes post‐sepsis are limited, generalized microvascular peripheral dysfunction is well‐documented during sepsis (Hinshaw, 1996), and many sepsis patients also suffer from ischemic heart disease (Merx & Weber, 2007).

In ventilated patients with frank septic shock, impaired myocardial perfusion reserve in the acute state has been suggested from echo‐Doppler of the left anterior descending artery (Ikonomidis et al., 2014). However, in patients with sepsis and unaffected arterial blood pressure, coronary blood flow seems normal, with an increased myocardial oxygen extraction (Cunnion et al., 1986). As in peripheral circulation, these alterations can be attributed to disturbed autoregulation of blood flow or disturbed oxygen utilization (Herbertson et al., 1995; Powell et al., 1991). Otherwise, sepsis‐induced cardiac myocardial perfusion has mainly been deduced from animal experiments with endotoxin‐induced sepsis or septic shock (Habimana et al., 2020). In such models, coronary artery blood flow may even be increased if perfusion pressure is not affected (Ince, 2005; Levy et al., 2005; Rudiger & Singer, 2007), and even myocardial high energy phosphates may be preserved in acute sepsis‐related cardiodepression (Solomon et al., 1994; Van Lambalgen et al., 1994). In animal models of sepsis, myocardial autoregulation is disrupted (Lorigados et al., 2015), and while coronary blood flow of septic sheep can increase as long as the perfusion pressure remains stable (Di Giantomasso et al., 2003, 2005), it has in dogs been shown that myocardial perfusion becomes uneven with hyper‐perfusion of epicardial regions and hypo‐perfusion of endocardial (Groeneveld et al., 1991).

With the use of gadolinium‐contrast and sequences for determination of organ blood perfusion, magnetic resonance imaging (MRI) offers a noninvasive reference standard for organ blood flows. With this technique, we investigated cardiovascular function and left ventricle (LV) myocardial perfusion in patients admitted to hospital with sepsis and septic shock during initial recovery and 1–2 months after when the patients had fully recovered. In addition, we determined renal cortical, splenic, and hepatic blood flow changes. We examined the hypothesis that depression of myocardial and other vital organ blood microcirculatory flows may be seen in human survivors of sepsis and septic shock, even with preserved myocardial function.

## 2 MATERIALS AND METHODS

### 2.1 Study population

The study protocol was approved by the Copenhagen Ethics Committee (H‐18063652). Twenty‐one septic (*n* = 16) or septic shock patients (*n* = 5) (median age 66 [range 24–84] years; 9 women) admitted to Herlev Copenhagen University Hospital between October 2019 and September 2020 were included and completed at least one cardiac MRI scan. Patients were diagnosed with sepsis or septic shock based on standard criteria (Evans et al., 2021; Schlapbach et al., 2024; Singer et al., 2016) including the necessity to infuse noradrenaline to maintain a mean arterial blood pressure (MAP) above 65 mmHg if considered shocked. After having ascertained patients were capable of providing informed consent, patients were approached on ward‐rounds during their hospital stay and provided informed consent to participate in the study. Positive bacterial cultures included streptococci pneumoniae (*n* = 6), other streptococci (*n* = 4), eschericia coli (*n* = 5), haemophilus (*n* = 1), klebsiella pneumoniae (*n* = 1), and enterobacter (*n* = 1). Three patients had negative blood cultures but were determined to have sepsis on clinical grounds (Singer et al., 2016) and indeed these patients also improved on antibiotics. No patient had a known history of cardiovascular disease including ischemic heart disease, but some were known with chronic obstructive pulmonary disease (*n* = 3), arterial hypertension (*n* = 5), previous stroke (*n* = 2), or diabetes (*n* = 3). One patient had lymphoma, and one patient had localized prostate cancer.

Patients admitted to hospital with sepsis had an MRI scan performed when clinically stable. Patients in septic shock underwent an MRI scan within 8 h upon transfer from the intensive care unit to the medical ward. None of the patients received nor‐adrenaline treatment during the MRI scans. Of the 21 patients, 13 returned for a follow‐up MRI scan 1–2 months after hospital discharge, when the patients were considered to have fully recovered but well before any other disease may have intervened. The latter MRI scan was performed to determine the changes seen with early recovery from sepsis or septic shock as a control of what can be considered their baseline cardiovascular function and organ perfusion. Biomarkers were collected at multiple points during hospitalization, and on the day of the MRI scan.

### 2.2 Cardiovascular MRI protocol

Patients were scanned in the supine position in a 1.5 Tesla MRI scanner (Achieva, Philips, The Netherlands) using a dedicated cardiac coil. Biventricular and left atrial (LA) volumes and biventricular systolic and diastolic functions were assessed from balanced steady‐state free precession cine imaging sequences (TR/TE 4.2 ms/1.7 ms, flip angle 60°, slice thickness 8 mm (2 mm interslice gap), field of view 320 × 320 mm, matrix 168 × 154, spatial resolution 1.9 × 2.1 mm, 30 phases per heartbeat) in a short‐axis stack covering the entire heart and, in addition, from 2‐, 3‐, and 4‐chamber views. Ascending and descending aortic areas were determined at end‐diastole and end‐systole from the transversal cine image at the level of the pulmonary artery and below the diaphragm immediately proximal to the left renal artery (Sørensen et al., 2020). Systolic (SAP) and diastolic (DAP) arterial blood pressures were determined by sphygmomanometry immediately before and immediately after the MRI scan, and the mean of these two determinations was used for calculations.

**FIGURE 1:**
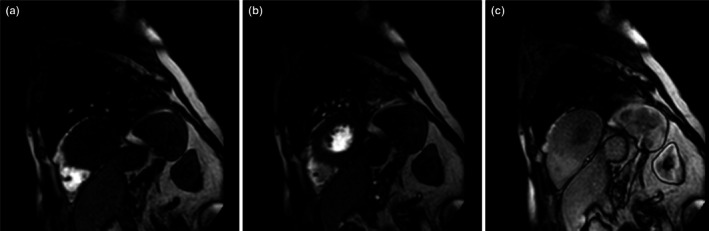
Representative MRI perfusion images illustrating contrast over time in the myocardium, liver, spleen, and kidneys following a gadolinium bolus injection via the right cubital vein. (a) Initial image with gadolinium contrast (bright white) only appearing in the lumen of the right ventricle. (b) Temporally intermediate image with gadolinium now visible in the lumen of the left ventricle and pulmonary vessels. (c) Final image showing gadolinium perfusion of the myocardium, liver, spleen, and kidneys.

Myocardial perfusion imaging was conducted using three short‐axis slices (basal, mid‐ventricular, and apical) with a perfusion sequence (TR/TE/flip angle 2.5 ms/1.3 ms/50°, slice thickness 12 mm, (5 mm interslice gap), field of view 350 × 350 mm, matrix 144 × 160, spatial resolution 2.8 × 3.2 mm). For contrast enhancement, via a large cubital vein, 0.1 mmol/kg of gadoterin acid (Dotarem®) followed by 20 mL of saline was administered intravenously at a rate of 2 mL/s by an automatic infusion pump (Sørensen et al., 2020). As previously validated, the perfusion sequence scan‐planes initially planned for the myocardial assessment also allowed for the determination of liver, spleen, and renal cortical blood flows (Figure 1; Kyhl et al., 2016).

Additionally, after 10 min, TI‐scout images were obtained to determine the inversion time that “nulled” the myocardial signal. Late gadolinium enhancement images were acquired in three LV short‐axis slices using an inversion recovery sequence at least 5 min from the injection of the gadolinium contrast (TR/TE/flip angle 2200 ms/60 ms/30°, slice thickness 12 mm with 5 mm interslice gaps, field of view 320 × 320 mm, matrix size 256 × 256, spatial resolution 1.5 × 1.9).

### 2.3 Cardiovascular MRI analysis

CMR scans were analyzed with third‐party software (Circle Cardiovascular Imaging Inc., Calgary, Canada, version 6.0.2). Epi‐ and endocardial borders were semi‐automatically traced in all phases in the short axis cine stack to derive LV and RV end‐diastolic volume (EDV), end‐systolic volume (ESV), stroke volume (SV), ejection fraction (EF), LV mass, and the derived parameter LV and right ventricle (RV) peak‐filling (PFR) and peak‐ejection (PER) rates (Kyhl et al., 2021). In addition, the maximal and minimal left (LA) and right atrial (RA) volumes were determined at ventricular end‐systole and ‐diastole using monoplane imaging for the right atrium and biplane imaging for the left atrium, based on 4‐ and 2‐chamber cine views. Perfusion sequences were analyzed for the myocardium, right renal cortex, spleen, and liver by placing regions of interest (ROIs) for each organ (Philips Intellispace Portal, Philips Medical Systems, Best, The Netherlands, version 9.0) to assess blood flow dynamics of each organ (Kyhl et al., 2016). As semiquantitative measures of organ blood flow (Panting et al., 2001; Panting et al., 2002; Schwitter et al., 2001; Kellmann & Arai, 2012; Ramachandran et al., 2024; Wu & Zhang, 2024) for each organ, we determined the time of onset of the gadolinium‐contrast signal (in sec.), the time to peak gadolinium‐contrast signal (in sec.), and the organ wash‐in rate of gadolinium‐contrast (in s^−1^). We consider the wash‐in rate the closest semiquantitative measure of blood flow but consider the combined measures of gadolinium‐contrast onset time, time to peak signal intensity, and wash‐in rates (Panting et al., 2001; Panting et al., 2002; Schwitter et al., 2001; Kellmann & Arai, 2012; Ramachandran et al., 2024; Wu & Zhang, 2024).

### 2.4 Calculations and statistical analysis

The MAP was determined as the sum of the DAP and 1/3 of the pulse pressure (the difference between the SAP and DAP). Cardiac output (CO) was determined as the product of heart rate (HR) and the LV stroke volume (LVSV). The rate‐pressure product (RPP) was determined as the product of HR and SAP as a measure determining LV myocardial energy demand (Gobel et al., 1978), and myocardial perfusion measures were related to RPP. Total peripheral resistance (TPR) was determined by dividing MAP by the CO. Volumes were indexed to the body surface area as determined from Mosteller's formula. The central blood volume, that is, the blood volume encompassed by the pulmonary circulation, was determined by Hamilton's technique from the product of the LVSV and the perfusion‐sequence‐determined gadolinium pulmonary transit time (Mijacika et al., 2017). Aortic distensibility was determined from the relative increase in aortic area with systole divided by the pulse pressure (Kyhl et al., 2021).

Statistical analysis was performed using SPSS statistics 29 (IBM, Armonk, New York, USA) to assess differences in cardiac function, perfusion, and biomarker levels between the initial and follow‐up MRI scans. For normally distributed variables and only two timepoints, a paired *t*‐test was applied. With three or more timepoints, ANOVA was applied to evaluate temporal changes, and a paired *t*‐test was conducted between the first and last timepoints if the ANOVA was significant. For non‐normally distributed variables, log transformation was performed, followed by a reassessment of normality with Shapiro–Wilk's test. If the log‐transformed data met normality criteria, the analysis proceeded as outlined above. With non‐normally distributed variables with more than two timepoints, changes with time were evaluated with Friedman's test, and changes with only two timepoints were analyzed with Wilcoxon's signed‐rank test. For all statistical tests, a significance level of 0.05 was chosen. This study was considered exploratory, and therefore we did not control for multiple comparisons with, for example, a Bonferroni analysis. Organ perfusion was judged on the basis of the combined changes to time of arrival of contrast, time to peak contrast perfusion, and wash‐in rates of contrast. An increased time of arrival or an increased time to peak of contrast signify a lowered blood flow, as does a lowered wash‐in rate of contrast (Li et al., 2024). Setup and data are reported according to STROBE (https://www.equator‐network.org/wp‐content/uploads/2015/10/STROBE_checklist_v4_cross‐sectional.pdf).

## 3 RESULTS

Paraclinical and MRI‐derived parameters are presented in Tables 1, 2, 3. With antibiotic treatment, during the first week of hospitalization, temperature and leucocytes normalized, and the hs‐CRP decreased; albeit it had not normalized on the first MRI day (69 ± 54 mmol/L, normal <10 mmol/L; Table 1). The SAP, MAP, and DAP remained stable throughout the hospitalization period, albeit in the five septic shock patients initially aided by noradrenaline infusion (median 3 (range 2–15) days of noradrenaline support). On the first MRI day, patients with septic shock had stabilized and were not given noradrenaline. In comparison with full recovery, HR decreased from 97 ± 21 bpm at admission to 77 ± 11 bpm on the first MRI day (*p* < 0.01). With maintained SAP and a slightly numerically higher HR, the RPP was elevated at initial recovery in comparison with the fully recovered situation (Table 2). At 2.2 ± 2.4 mmol/L, the s‐lactate levels were slightly increased at admission but had near‐normalized on the first MRI day, and pH values and hemoglobin were normal during hospitalization. The arterial oxygen saturation and carbon dioxide tension were initially affected (at 75% admission time, SaO_2_ was 91 ± 9% and PaCO_2_ was 4.9 ± 0.5 kPa) but both had normalized before the first MRI day.

**TABLE 1:** Arterial blood pressures, heart rate, and biochemical values at admission and during hospitalization and the magnetic resonance imaging day (MRI; “early recovery”) for 21 patients admitted to hospital with sepsis (*n* = 16) or septic shock (*n* = 5) and on the second MRI day (“full recovery”; *n* = 16).

Hospitalization (% of duration before first MRI scan day)	Day 0	25%	50%	75%	MRI day 1	MRI day 2	*p* Value MRI day 1 vs. 2	Effect size	Power
SAP (mmHg)	127 ± 33	129 ± 25	131 20	129 ± 23	127 ± 18	126 ± 15	0.94	0.01	0.06
DAP (mmHg)	68 ± 15	69 ± 14	71 ± 12	70 ± 13	72 ± 12	74 ± 11	0.68	0.16	0.16
MAP (mmHg)	88 ± 19	89 ± 17	91 ± 13	90 ± 16	91 ± 13	92 ± 9	0.83	0.33	0.52
Pulse pressure (mmHg)	59 ± 24	60 ± 17	60 ± 17	58 ± 15	55 ± 12	52 ± 18	<0.01	0.87	0.99
HR (bpm)	93 ± 23	85 ± 19	81 ± 15	76 ± 19	78 ± 12*	74 ± 11	<0.01	0.51	0.94
RPP (mmHg bpm)	12,422 ± 3432	11,145 ± 3180	10,610 ± 1563	9374 ± 2790	9985 ± 172	9771 ± 1649	0.02	0.48	0.91
S‐Na (mmol/L)	138 ± 6	142 ± 5	142 ± 4	133 ± 31	140 ± 3	—	<0.01	0.48	0.91
S‐K (mmol/L)	3.5 ± 0.7	3.5 ± 0.5	3.5 ± 0.5	3.5 ± 0.5	3.6 ± 0.4	—	0.41	0.28	0.44
S‐Creatinine (umol/L)	113 ± 52	101 ± 41	87 ± 34	73 ± 27	71 ± 24*	—	0.08	0.57	0.88
Hs‐CRP (mmol/L)	200 ± 123	206 ± 122	163 ± 107	102 ± 65	69 ± 54^#^	—	<0.01	1.1	1
ALAT (u/L)	99 ± 188	91 ± 136	90 ± 120	82 ± 116	78 ± 125	—	0.79	0.18	0.2
LDH (u/L)	348 ± 314	365 ± 306	321 ± 209	281 ± 153	265 ± 144	—	0.01	0.77	0.99
S‐Carbamide (mmol/L)	9.2 ± 6.7	8.7 ± 5.5	8.4 ± 5.4	6.8 ± 3.6	5.6 ± 2.5^#^	—	0.07	0.44	0.66
S‐leukocytes (cells/μL)	13.3 ± 8.8	13.1 ± 9.9	11.8 ± 8.2	9.9 ± 3.6	9.6 ± 3.3	—	<0.01	0.72	0.99
S‐hemoglobin (mmol/L)	7.4 ± 1.3	7.1 ± 1.1	6.8 ± 1.1	6.8 ± 1.1	7.1 ± 1.1	—	0.03	0.59	0.98
S‐lactate (mmol/L)	2.1 ± 2	1.7 ± 0.9	1.3 ± 0.4	1.2 ± 0.4	1.1 ± 0.4*	—	<0.01	0.69	0.99
S‐HCO_3_ (mmol/L)	23.4 ± 3.1	23.9 ± 2.7	25 ± 2.3	25.4 ± 2.6	25 ± 2.5	—	0.78	0.18	0.21
S‐pH	7.43 ± 0.04	7.45 ± 0.04	7.45 ± 0.05	7.44 ± 0.04	7.47 ± 0.04	—	0.05	0.46	0.89

*Note*: Values are reported on Day 0, and 25%, 50%, 75% of the time before the first MRI scan. Values are means ± SD.

Abbreviations: ALAT, serum alanine aminotransferase; DAP, diastolic arterial pressure; HR, heart rate; hs‐CRP, highly sensitive C‐reactive protein; LDH, serum lactate dehydrogenase; MAP, mean arterial pressure; RPP, rate‐pressure product; SAP, systolic arterial pressure; S‐HCO_3_, serum bicarbonate; S‐K, serum potassium; S‐Na, serum sodium.

* *p* < 0.01 as compared to Day 0.

^#^ *p* < 0.05 as compared to Day 0.

**TABLE 2:** Cardiac volumes and cardiac function as determined by magnetic resonance imaging in 21 patients during and 1 month after hospital admission for severe infection.

	All 21 patients initial recovery	13 patients initial recovery	Rescanned full recovery	*p* Value rescanned pts.	Effect size	Power
LVESV_(I)_ (mL/m^2^)	29 ± 10	29 ± 8	29 ± 8	0.96	0.07	0.06
LVEDV_(I)_ (mL/m^2^)	73 ± 18	76 ± 15	70 ± 13	0.16	0.4	0.27
LVSV_(I)_ (mL/m^2^)	44 ± 15	48 ± 10	41 ± 7	0.05	0.64	0.57
RVESV_(I)_ (mL/m^2^)	33 ± 10	33 ± 10	31 ± 10	0.01	0.31	0.18
RVEDV_(I)_ (mL/m^2^)	73 ± 23	78 ± 23	69 ± 21	0.17	0.45	0.32
RVSV_(I)_ (mL/m^2^)	39 ± 16	43 ± 15	38 ± 13	0.76	0.30	0.17
Heart rate (bpm)	78 ± 15	75 ± 9	72 ± 12	0.63	0.14	0.07
CI (L/min/m^2^)	3.4 ± 2	3.9 ± 1.0	3.1 ± 0.7	0.02	0.72	0.66
LVEF (%)	60 ± 12	59 ± 3	59 ± 6	0.12	0.14	0.08
RVEF (%)	52 ± 13	56 ± 10	57 ± 7	0.83	0.06	0.05
TPR (mmHg/L/min)	13 ± 4	13 ± 3	17 ± 3	<0.01	0.87	0.82
Central blood volume (mL)	692 ± 298	603 ± 231	572 ± 192	0.73	0.22	0.13
LVPFR (mL/s)	773 ± 453	794 ± 410	518 ± 228	0.05	0.61	0.53
LVPER (mL/s)	738 ± 374	853 ± 478	543 ± 163	0.04	0.66	0.59
RVPFR (mL/s)	598 ± 263	614 ± 273	665 ± 486	0.89	0.04	0.05
RVPER (mL/s)	534 ± 254	677 ± 266	614 ± 273	0.97	0.01	0.05
RA_max (I)_ (mL)	31 ± 14	33 ± 16	27 ± 7	0.25	0.42	0.29
RA_min (I)_ (mL)	17 ± 10	18 ± 12	15 ± 7	0.73	0.31	0.18
LA_max (I)_ (mL)	42 ± 8	43 ± 15	33 ± 14	0.01	0.62	0.54
LA_min (I)_ (mL)	21 ± 8	20 ± 8	17 ± 6	0.09	0.39	0.26
Ascending AD (Δ%‐point)	0.086 ± 0.17	0.087 ± 0.18	0.091 ± 0.30	0.86	0.13	0.08
Descendending AD (Δ%‐point)	0.009 ± 0.27	0.024 0.27	0.070 ± 0.25	0.91	0.05	0.03

*Note*: Values are means ± SD.

Abbreviations: CI, cardiac index; LA LVED, left atrium volume at left ventricular end‐diastole; LA LVES, left atrium volume at left ventricular end‐systole; LVEDVI, left ventricular end‐diastolic volume index; LVEF, left ventricular ejection fraction; LVESVI, left ventricular end‐systolic volume index; LVPER, left ventricular peak ejection rate; LVPFR, left ventricular peak filling rate; LVSVI, left ventricular stroke volume index; RA LVED, right atrium volume at left ventricular end‐diastole; RA LVES, right atrium volume at left ventricular end‐systole; RVEDVI, right ventricular end‐diastolic volume index; RVEF, right ventricular ejection fraction; RVESVI, right ventricular end‐systolic volume index; RVPER, right ventricular ejection rate; RVPFR, right ventricular peak filling rate; RVSVI, right ventricular stroke volume index; TPR, total peripheral resistance.

**TABLE 3:** Left ventricular (LV) myocardial, renal cortical, spleen, and hepatic blood perfusion dynamics as determined by magnetic resonance imaging with gadolinium‐contrast perfusion sequences in 21 patients during and 1–2 months after hospital admission for severe infection. Values are means ± SD.

	All 21 patients initial recovery	13 patients initial recovery	Rescanned full recovery	*p* Value for rescanned	Effect size	Power
LV myocardium	*n* = 17	*n* = 13	*n* = 13			
Peak (s)	26 ± 15	29 ± 17	38 ± 16	0.03	0.67	0.61
Peak/RPP	0.0039 ± 0.003	0.0031 ± 0.002	0.0039 ± 0.002	0.13	0.44	0.31
Onset (s)	8 ± 5	12 ± 11	33 ± 20	0.02	0.78	0.40
Onset/RPP	0.0012 ± 0.001	0.0012 ± 0.001	0.0035 ± 0.002	0.01	0.81	0.77
Wash‐in rate (s^−1^)	25 ± 21	23 ± 22	14 ± 14	0.07	0.57	0.47
Wash‐in rate/RPP	0.004 ± 0.004	0.0025 ± 0.003	0.0014 ± 0.001	0.76	0.54	0.43
Renal	*n* = 9	*n* = 7	*n* = 7			
Peak (s)	28 ± 14	29 ± 17	26 ± 11	0.85	0.07	0.05
Onset (s)	11 ± 7	16 ± 13	14 ± 9	0.27	0.46	0.18
Wash‐in rate (s^−1^)	31 ± 17	32 ± 18	26 ± 13	0.57	0.23	0.08
Spleen	*n* = 15	*n* = 13	*n* = 13			
Peak (s)	32 ± 13	29 ± 13	39 ± 17	0.87	0.04	0.05
Onset (s)	12 ± 6	16 ± 12	15 ± 13	0.66	0.13	0.08
Wash‐in rate (s^−1^)	28 ± 22	30 ± 22	30 ± 22	0.97	0.01	0.61
Liver	*n* = 17	*n* = 12	*n* = 12			
Peak (s)	48 ± 10	48 ± 13	48 ± 12	0.87	0.04	0.05
Onset (s)	16 ± 9	19 ± 8	17 ± 7	0.62	0.014	0.07
Wash‐in rate (s^−1^)	13 ± 9	13 ± 10	22 ± 16	0.03	0.68	0.05

With initial recovery, although LVEF was not increased, left ventricular peak ejection rate (LVPER) was, and both HR and LVSV and consequently the cardiac index (CI, by approx. 27% in comparison with the full recovery) were all elevated (all *p* < 0.05). In comparison with full recovery, TPR was lowered at initial recovery. Right heart volumes and functional parameters were not significantly affected by sepsis. The central blood volume was normal and unchanged between initial and full recovery, but the indexed LA maximal volume was slightly elevated with initial recovery (Table 2). There were no significant changes to ascending and descending aortic distensibilities between time points, and aortic distensibilities were not different in patients who had recovered from septic shock with noradrenaline infusion (data not shown).

As compared to the full recovery, the gadolinium‐contrast related onset of LV myocardial perfusion (12 ± 11 vs. 33 ± 20 s; *p* = 0.02) and the peak perfusion of LV myocardial perfusion both came earlier (29 ± 17 s vs. 38 ± 16 s; *p* = 0.03) at the initial recovery as compared to late recovery (Table 3). Also, the overall LV myocardium gadolinium‐contrast wash‐in rate tended to be higher at the initial recovery than during full recovery (23 ± 22 vs. 14 ± 14 s^−1^; *p* = 0.07). These changes seemed related to RPP (Table 3). There were no significant LV myocardial late gadolinium hyperenhancements in any patients, neither at early nor late recovery MRI scans. Renal, spleen, and hepatic onset of gadolinium‐contrast‐perfusion, time to peak‐perfusion, and wash‐in rates were all comparable between the initial recovery and the full recovery, except for the wash‐in rate of the liver that showed a significant increase with full recovery to 22 ± 16 s^−1^ at follow‐up from a value of 13 ± 10 s^−1^ at initial recovery (*p* = 0.03).

**FIGURE 2:**
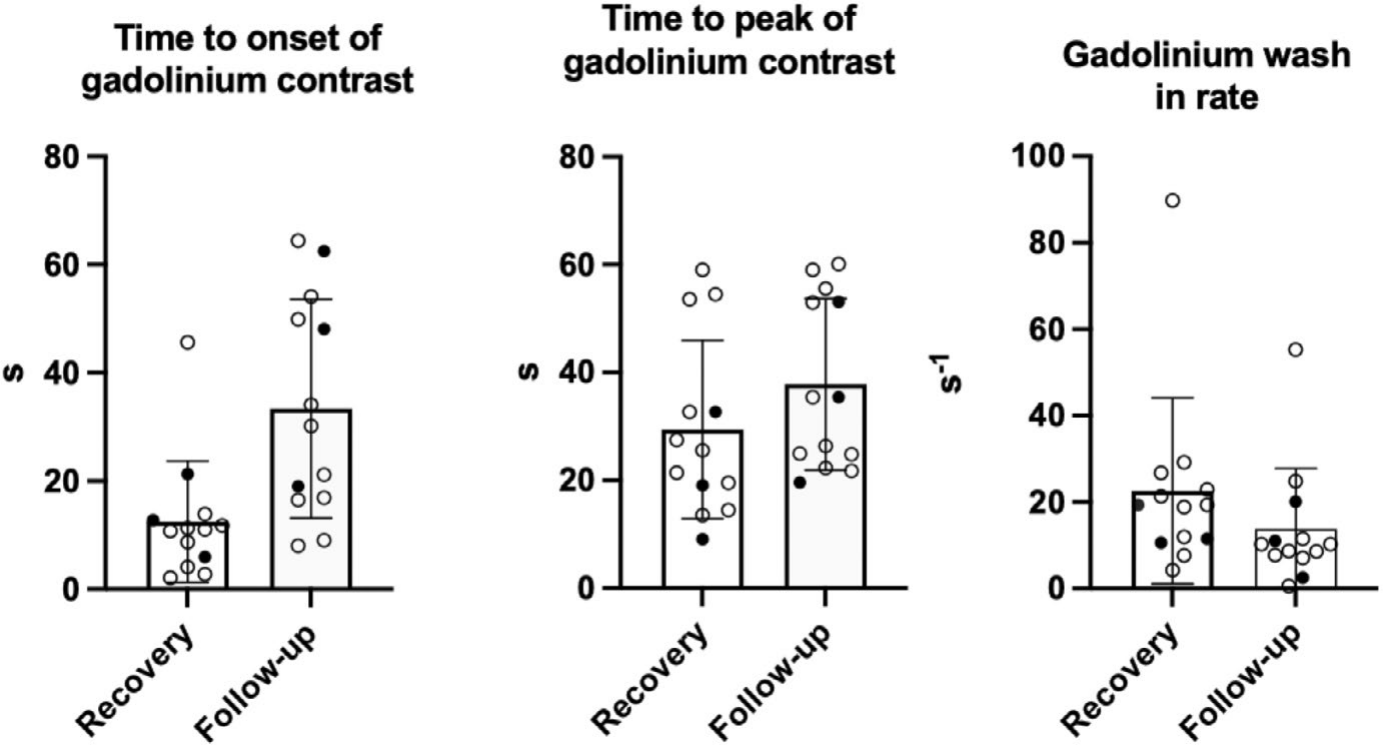
Left ventricular myocardial perfusion as determined from time to onset of magnetic resonance imaging gadolinium‐contrast perfusion sequences, time to peak of perfusion, and gadolinium wash‐in rate at initial recovery and after full recovery (1–2 months) after hospital admission for severe infection in 21 patients admitted to hospital with sepsis or septic shock. Open circles, patients with sepsis. Filled circles, patients with septic shock. Vertical bars represent means ± standard deviation.

Analysis of myocardial perfusion between the group of septic shock patients (*n* = 5) and the group of sepsis patients (*n* = 16) showed a numerically later onset of myocardial perfusion (13 ± 8 vs. 8 ± 5 s; *p* = 0.31), a shorter time to peak perfusion (20 ± 12 vs. 28 ± 15 s; *p* = 0.14), and a 50% lower wash‐in rate (14 ± 5 vs. 29 ± 25 s^−1^; *p* = 0.43) in patients who had recovered from septic shock, although none of these differences reached statistical significance (Table 3; Figure 2). No differences were noted for other organ blood flows between patients with sepsis and septic shock. Furthermore, organ perfusion parameters, including hepatic measures, were not related to biomarkers.

## 4 DISCUSSION

In a cardiovascular MRI study applying perfusion sequences, in 21 patients admitted to hospital with sepsis or septic shock, myocardial blood flow was maintained or even high in the initial recovery phase in comparison with measurements performed after 1–2 months, when patients had fully recovered. In contrast, the overall hepatic blood flow was lowered during initial recovery as determined from the wash‐in rate of gadolinium contrast, while the time to contrast arrival and time to peak hepatic contrast perfusion were unchanged. Blood flow of the spleen and the renal cortex was not affected by severe infection. A higher left atrial volume with initial recovery suggested an initial high preload for the left ventricle, but the central blood volume was comparable to levels observed at follow‐up. The increase in myocardial blood flow with infection was consistent with the higher RPP in addition to the related 27% increase in CO and higher LVPER and LVPFR in comparison with the follow‐up. No gadolinium‐late enhancement of the myocardium was noted, and hence severe infection does not seem to lead to areas of myocardial fibrosis.

This study, which utilized the noninvasive reference technique of gadolinium‐contrast MRI perfusion sequences, did not show any significant microvascular perfusion problems of the myocardium with acute severe infection, and in fact all patients had quickly recovered with normal or even supra‐normal systolic and diastolic ejection and filling parameters. Also, this study could not demonstrate any late gadolinium hyperenhancement changes to the myocardium. Our findings are best in line with the notion that if severe infection is well treated, such is not associated with cardiovascular compromise or indeed with changes to the myocardial perfusion in the immediate aftermath of infection. The slightly increased myocardial blood flow seen with sepsis corresponds to the slightly elevated RPP. The increasingly noted problems of later heart failure therefore cannot be attributed to immediate damage to the myocardium or indeed to microvascular dysfunction. However, in the few septic shock patients we studied, numerically lower wash‐in rates of gadolinium contrast were observed. It is increasingly speculated that later heart failure, if such may occur, may relate to for example, a continually elevated inflammatory response. For example, elevated hs‐CRP has been reported up to 5 years after a septic episode (Felici et al., 2021). It is known that a generally elevated hs‐CRP level is an independent risk factor for not only later septic episodes (Wang et al., 2013), but also later atherosclerosis (Kattula et al., 2017) and indeed clinically verified ischemic heart disease (Arnett et al., 2019) and heart failure (He et al., 2023; Suleiman et al., 2006). Hs‐CRP remained markedly higher than normative values (69 ± 54 mmol/L vs. normal <10 mmol/L).

Hepatic dysfunction is common in sepsis and is an independent arbiter of sepsis‐related mortality by intensifying systemic inflammation (Beyer et al., 2022; Foster & Kellum, 2023; Jacobi, 2022; Kellum & Ronco, 2023). As judged from the wash‐in rate of gadolinium contrast, our study suggested a possibly lowered hepatic blood perfusion during initial recovery, but ALAT values were normal, suggesting that oxygen flow to hepatocytes was not critically lowered. The gadolinium‐contrast wash‐in rate of the liver is, however, influenced by both the hepatic artery and portal vein inflows. The valve‐less portal vein serves a low‐pressure/low‐resistance system that provides partially deoxygenated blood from the intestinal bed to the liver, accounting for 70% of hepatic blood supply (Lautt, 1981). In general, oxygen delivery to hepatocytes does not depend on the proportion of portal versus arterial blood flow (Lutz et al., 1975), and as oxygen extraction changes with variations in demand and supply, the hepatocellular oxygen extraction can approach 100% (Lautt, 1981). With the applied technique using a ROI representing hepatic tissue per se, we cannot discern arterial from portal venous supply, but it is well established that the hepatic artery blood flow is prominently autoregulated (“hepatic artery reflex”, Eipel et al., 2010; Lautt, 2007; Keramida et al., 2020). Consistent with this, the time to arrival and peak values for gadolinium contrast were unchanged. Hence, the lowered hepatic wash‐in rate likely reflects an initial decrease in portal vein flow and thereafter fully recovery of the total hepatic perfusion with improvement of the portal vein flow, only after the patient has fully recovered. Studies suggest that the liver's microcirculation may also suffer from increased inflammatory cytokines and endothelial damage (Beyer et al., 2022; Kasper et al., 2020; Strnad et al., 2017), making it difficult to maintain adequate blood flow, especially in cases where vasopressors known to decrease splanchnic blood flow (and hence bringing splanchnic venules to a lower distension pressure) are required for maintaining an overall normal central blood volume and a normal arterial blood pressure (Rowell & Rowell, 1993).

As was the case for the myocardium, our study results did not document any significant changes to renal cortical or splenic perfusions. The kidneys are highly susceptible to sepsis‐induced acute kidney injury, and this has previously been suggested to be due to impaired renal perfusion at the microvascular level (Manrique‐Caballero et al., 2021; Peerapornratana et al., 2019). Even though our study did not find any significant changes, studies using cine phase‐contrast MRI reveal that even when systemic hemodynamics appear stable, renal cortical perfusion is frequently compromised, resulting in inadequate filtration, and accumulation of serum toxins (Prowle et al., 2012). This impaired perfusion can be further exacerbated by the use of vasopressors, which are essential for maintaining systemic arterial blood pressure but can restrict renal blood flow (McDonald et al., 2024). However, vasopressor but did not seem to affect the renal blood flow in this study, albeit smaller changes may go unnoticed due to low statistical power.

Recent, albeit small, studies have suggested that RV function may serve as a more sensitive prognostic marker for mortality than LV in septic shock (Bansal et al., 2023; Lanspa et al., 2021), and RV dysfunction may be more strongly associated with both long‐term and short‐term mortality (Vallabhajosyula et al., 2021). For example, in 25 septic shock patients, RVEF was higher in survivors than in non‐survivors (Liu et al., 2000), and a reduction in RVEF was noted during early recovery, indicating that RV dysfunction may persist longer than LV dysfunction in septic shock patients. Though our study also only has a limited number of patients, our study, with right heart volumes measured by the reference technique of MRI (Liu et al., 2000), did not show changes in RVEF from early recovery to follow‐up and indeed also found normal RV volumes and function in early recovery.

### 4.1 Strengths and limitations

It is a strength of our study that we determined myocardial and other organ blood flows with the noninvasive reference technique of cardiovascular MRI and that patients were their own controls. However, our study has important limitations. It is noteworthy that we were unable to study patients during the acute phase of sepsis or septic shock. All patients included in the study had normal arterial blood pressures even without vasopressors at the time of scan. As a result, the study is essentially underpowered in these respects, and we cannot draw conclusions about what happens in the acute stage or in patients experiencing septic shock. Another limitation is that our control situation, by necessity, was based on the period when patients were apparently fully recovered. We did not have data on, for example., organ blood flow prior to hospital admission, making it impossible to directly compare pre‐admission and post‐recovery states. We therefore relied on the 1–2‐month follow‐up scan to be representative of the normal preadmission cardiovascular situation, but this assumption may not hold. Organ blood flow could have been significantly altered by the septic episode or hospital admission and may generally have been lower than what would have been observed pre‐sepsis. The patients with septic shock demonstrated a numerical tendency for having lower myocardial blood flows with acute sepsis and also were patients in the lower end of the recovery myocardial perfusion spectrum (Figure 2). Such may be consistent with a tendency for patients with either low (albeit still normal) myocardial perfusions to more easily become shocked if septic or for such patients to eventually develop microvascular dysfunction. One further limitation is the exploratory nature of our study, with a high number of physiologically meaningful parameters measured. We acknowledge that the slightly lowered hepatic wash‐in rate with initial recovery, here interpreted as resulting from a lowered portal vein flow, may also represent a type I statistical error.

## 5 CONCLUSION

In hospitalized patients with severe systemic bacterial infection, where arterial blood pressure and pH are normalized, myocardial blood flow is slightly elevated relative to the increased rate–pressure product and hence myocardial oxygen demand. Our study suggests that any possible later increased risk of heart failure and ischemic heart disease in these patients is not due to immediate detrimental changes in myocardial microvascular perfusion during acute infection. Rather, with effective treatment, severe infection does not compromise myocardial microvascular integrity in the short term. Future research should focus on understanding these long‐term risks and examining organ‐specific responses to sepsis to elucidate the mechanisms underlying post‐septic cardiovascular disease, and our results suggest more studies should be performed in patients who have recovered from notably septic shock.

## ETHICS STATEMENT

The study protocl was approved by the Copenhagen Ethics Committee (H‐18063652).

## Data Availability

Data will be shared on reasonable request.
